# Environmental Exposures in the Context of Child Care

**DOI:** 10.1289/ehp.121-a160

**Published:** 2013-05-01

**Authors:** Nate Seltenrich

**Affiliations:** Nate Seltenrich covers science and the environment from Oakland, CA. His work has appeared in *High Country News*, *Sierra*, *Earth Island Journal*, the *San Francisco Chronicle*, and other local and national publications.

Just beyond the front door of the Montessori School at Five Canyons, a square glass-walled foyer is brimming with verdant houseplants in clay pots. Garden sculptures and glazed ceramic art are interspersed throughout. Above it all floats the looped sound of softly chirping birds. This lush tableau provides a fitting transition between the world outside and the carefully controlled atmosphere within, where child care director Meher Van Groenou has made environmental health one of her top priorities.

The school serves 120 toddlers, preschoolers, and kindergarteners in Castro Valley, California.^1^ Within its five classrooms, most toys and utensils are made of wood, glass, or stainless steel. Ample windows welcome natural light and permit cross-ventilation on warmer days. The carpets contain no glue, nor does the tongue-and-groove wood flooring.

Van Groenou helped design the building 11 years ago, drawing from her experience seeking to provide a healthy home for her own children. Green construction was by then already being embraced in California’s residential and commercial sectors, and in many schools—but not child care centers. There were few child care–specific resources to support her, no local standards to lead her, and hardly any other centers to offer a model.

Research has proven that infants and toddlers, who spend more time on the floor and experience the world with their hands and mouths, are not merely in closer contact with many indoor pollutants^2^ but also more sensitive to them.^3^ Yet environmental health standards in child care settings nationwide—which can include not just centers but also private homes, workplaces, universities, and places of worship—still lag behind those of schools, where children are older, larger, and somewhat less susceptible to environmental exposures. Unlike with more uniformly regulated schools, child care licensing, permitting, and oversight occur on a variety of levels, resulting in a fractured regulatory landscape.

A host of other factors, many of them specific to child care, contribute to the challenge. For example, licensing guidelines and quality rating systems—which often emphasize infection control and cleanliness—can steer centers toward bleach or other potentially toxic sanitizers and disinfectants that are now recognized as asthma triggers,^4^ says Ellen Dektar of the Alameda County Childcare Planning Council; even the Five Canyons center disinfects with diluted bleach. For similar reasons, other facilities may choose pesticides over prevention-based approaches to pest management.

Tight budgets and slim profit margins in the child care industry leave little room for pricier green products and hazard mitigation or removal. Meanwhile, licensed child care providers must meet growing requirements pertaining to disaster preparedness and care of children with special needs, says Hester Paul, national director of Eco-Healthy Child Care® (EHCC),^5^ a green child care endorsement and training program.

Teaching child care staff—who may be poorly educated, nonfluent in English, and/or already challenged by the demands of their jobs—about environmental exposures “can be a formidable task,” says Vickie Leonard, a researcher at the University of California (UC), San Francisco, who is working to develop child care–specific resources on green cleaning, sanitizing, and disinfecting.

The same can be true for credentialed child care directors, says Karen Teliha, community and environmental health coordinator for Indiana’s 5-Star Childcare program,^6^ the nation’s only comprehensive statewide environmental health certification program for such facilities. “For most child care providers, environmental health is a newer area,” Teliha says. “Educating them about pest control and proper pesticide usage, that’s not something that’s necessarily taught when you go to become a child care director.”

In each case, the first step is to learn more about what, exactly, infants and toddlers are being exposed to. But the deeper one looks, the more complex it gets.

## New Findings

Even the field’s brightest minds say they need a better grasp on the issue. After all, the incredible diversity of child care settings is surpassed only by their sheer numbers. Approximately 330,000 child care centers serve 11.7 million children nationwide,^7^ or 45% of all children aged 5 years and younger.^8^ Gaining access to such facilities—and trust from the providers—can be a challenging proposition for researchers.

Gleaning environmental exposure data from this vast subject area has been the aim of a small handful of studies to date, which have begun to provide a preliminary foundation for ongoing educational and regulatory efforts. Most recently, UC Berkeley researcher Asa Bradman, a pioneer in the field who began his work nearly a decade ago after discovering high levels of lead inside his daughter’s child care facility, led a groundbreaking study on environmental quality in early childhood education environments.^9^

Bradman’s team used a variety of sampling and analytical methods to test the indoor air and floor dust of 40 child care facilities, including centers and home-based programs, in California’s Alameda and Monterey counties. Their report, released in April 2012 by the California Air Resources Board, which funded the work, is the first and only of its kind to measure and analyze a broad spectrum of pollutants inside U.S. child care centers.^10^ Pollutants of interest included volatile organic compounds (VOCs), particulate matter, pesticides, flame retardants, phthalates, and perfluorinated compounds.

The study found that the levels of most pollutants were similar to those measured in California schools and residences. However, a few areas of particular concern emerged. In 35 of the 40 facilities, formaldehyde levels exceeded California’s strict 8-hour and chronic reference exposure levels.^10^ The chemical—a known carcinogen^11^ associated with acute irritation of the eyes, skin, and respiratory tract^12^—is used extensively in composite and pressed-wood furniture and construction materials. It also is found in carpets, carpet pads, paints and coatings, furniture fabrics, draperies, personal care products, and permanent-press clothing.^13^ All these items are common within the child care setting.

In one-third of the centers, levels of acetaldehyde,^14^ a related compound, exceeded the reference concentration set by the U.S. Environmental Protection Agency (EPA) for respiratory and irritant effects. In most facilities, levels of other VOCs including aldehydes, chloroform, benzene, and ethylbenzene exceeded child-specific “safe harbor levels” that were computed by the report authors based on California Proposition 65^15^ guidelines. (“Safe harbor” refers to exposure thresholds below which adverse effects are unlikely to occur.)

The study also found that indoor concentrations of coarse particulate matter measured over periods of 8–10 hours exceeded the 24-hour California Ambient Air Quality Standard^16^ in nearly half the facilities. And lead, which causes adverse developmental effects in children and for which no safe level of exposure has been determined,^17^ was detected in dust samples from 95% of the facilities.

Meanwhile, median levels of brominated flame retardants in dust were lower than levels reported in similar studies of residential environments. The authors speculated this may be a result of the frequent cleaning and vacuuming that occurs in child care facilities. However, they also found that in four of the centers, estimated exposures to two brominated flame retardants exceeded the U.S. EPA noncancer reference dose for children under the age of 1. And although pesticides were frequently detected in dust and in the air—more than half the facilities reported using them, with 45% using imprecise broadcast applications such as sprays—pesticide exposures did not exceed any existing health-based benchmarks.

The study’s results are nothing short of monumental, according to Paul: “What we need is a baseline, which is what [this] study really showed—What exposures are taking place in the child care setting?”

## Tip of the Iceberg

Still, Bradman’s findings only hint at what is yet to be learned. For example, although the team measured 39 known VOCs in the centers—most of which have no established health-based benchmarks—these measurements pointed to another approximately 130 VOCs that they weren’t looking for, chemicals with poorly understood individual, additive, and/or synergistic effects inside tiny bodies.^10^

The authors’ use of general-population guidelines to estimate safe harbor levels for children suggests that more work is also needed in risk assessment. And, Paul notes, the study’s risk assessment does not account for mixed exposures. Bradman says this will require the development of new standardized approaches.

**Figure:**
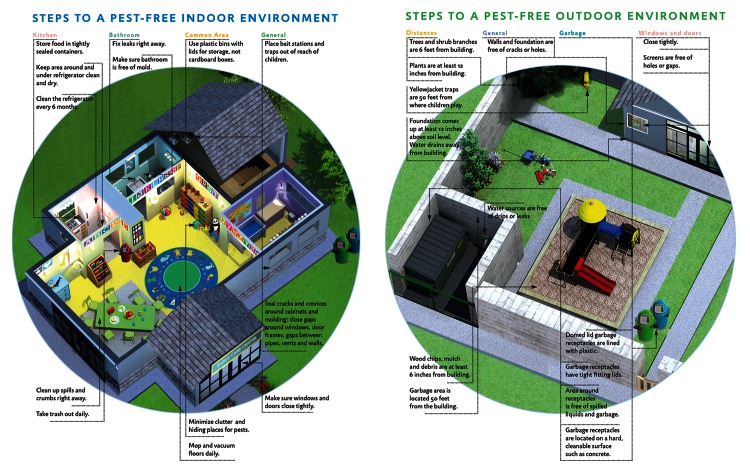
Prevention-based pest management is a key element of efforts to improve environmental health in child care settings. © UCSF California Childcare Health Program/http://www.ucsfchildcarehealth.org Copywriting: Victoria Leonard, RN, PhD; Design: Robin Brandes/http://www.robinbrandes.com; Illustration: Noa Kaplan/http://www.noapkaplan.com

Other future studies should be targeted toward identifying the actual sources of toxics, Bradman says. “There are a lot of indoor exposures that we need to learn more about,” he explains—for example, ultrafine particles that tend to spike during cooking times.^18^

The 2012 report builds on a limited body of work that offers some additional insight. The first large-scale effort came in 2003 from the U.S. EPA, in a report fittingly titled *First National Health Survey of Child Care Centers*.^19^ The study examined 168 child care centers across the country to calculate the prevalence of lead-based paint (estimated at 28%) and other lead hazards in child care centers nationwide.

In 2004 came another report, *Measuring Environmental Hazards in the Childcare Industry: Pesticides, Lead, and Indoor Air Quality*,^20^ prepared by environmental health and integrated pest management (IPM) advocate Phil Boise as a needs assessment for his GreenCare for Children program, a certification and training system. The study hinged on a survey administered to 748 child care providers in three Central California counties. It identified a number of areas of concern, especially around pesticide use, asthma triggers, and lead paint.

Like Bradman, Boise began investigating toxics in child care centers after making a troubling discovery of his own: One day while picking up his 11-month-old son from child care, he witnessed the provider spraying down an indoor Christmas tree with insect repellent as children slept nearby.

“It suddenly dawned on me that our most vulnerable population was being cared for by providers who needed some help making important decisions,” Boise says. “I knew that they needed more direction and that I as a parent needed a new mechanism to find care for my children.”

Bradman and colleagues continued their work with a 2008 survey of hundreds of California child care centers on their pest management practices.^21^ Among the results, 55% of the 637 respondents reported using pesticides, with 47% using sprays and foggers (as opposed to more targeted baits). More than half of these said they didn’t always notify parents and post warning signs when sprays and foggers were applied, as required under California’s Healthy Schools Act (HSA); one-fourth reported that they never did. “This survey was to gather info and support strategic planning for future outreach,” Bradman explains. Today, he says, “The California Department of Pesticide Regulation is supporting extensive outreach to child care providers to educate about pesticide use and the HSA.”

## Education and Certification

Such educational efforts may be paying off. In recent years, increased awareness of environmental health has begun to spread through the world of child care. A small handful of researchers, nonprofits, and public agencies scattered across the country—as well as early adopters like Van Groenou, who has delivered presentations on green child care to other Montessori schools—are beginning to change minds, and in some cases, policies.

“There’s a huge transformation going on right now in awareness within the child care community,” says Bradman. “We’re at a tipping point where there’s increasing demand for information and new products that are low-cost and can reduce environmental exposures.”

Paul shares Bradman’s enthusiasm. “What we’re seeing is a greater and greater awareness of the risk of environmental exposures causing short-term or long-term problems for children,” she says. “We have many child care, public health, and environmental health associations and organizations that believe in what we’re doing and want to support it.”

Indeed, the eight-year-old EHCC program is poised for rapid growth. The voluntary training and endorsement program, operated by Children’s Environmental Health Network, currently endorses more than 700 child care providers in 43 states and the District of Columbia, Australia, and Canada, serving more than 36,000 children.^22^ These facilities have agreed to comply with at least 24 items from a checklist of 30 free or low-cost steps to safeguard environmental health. As part of the program the providers agree to submit to an external inspection by the organization.

**In order to help meet the growing demand for information, Bradman and his colleagues have developed some recommendations for child care providers that they hope to publish eventually:**

1. Every child care program should have one person who is responsible for environmental health, a person who is educated on the issues, implements policies, and documents implementation in writing.
2. Every child care program should have written policies on purchasing and using products for pest management, cleaning, and similar activities.
3. Extensive local and regional training resources should be developed to assist child care providers on environmental health issues.
4. Child care providers should promote environmental health within the community, for example by encouraging parents to practice IPM and get their children tested for lead exposure.

Some of the nation’s leading child care entities are coming on board, Paul says. The General Services Administration, which offers child care to federal employees and other citizens at more than 110 centers nationwide, boasts that 91% of its centers are currently endorsed by EHCC. Children’s Creative Learning Centers, a private company that provides employer-sponsored child care, is also certifying its approximately 100 facilities. And Bright Horizons Family Solutions, one of the world’s largest child care services providers, is working to support all 775 of its centers in the United States, Britain, the Netherlands, Ireland, Canada, and India in attaining the EHCC endorsement, Paul says.

To complement its endorsement program, EHCC delivers in-person training to child care licensing staff, educators, and health consultants, who in turn train providers. To date it has trained approximately 600 child care professionals in 21 states. The program also offers its checklist, 16 fact sheets, and other resources for free online, and plans to translate some of its materials into Spanish.^5^

EHCC recently received three multiyear grants totaling $825,000, says Paul. “This support signifies the importance of protecting children from environmental health hazards in the child care setting,” she says. “Honestly, the program has just kind of snowballed.”

Other voluntary, pledge-based certification programs include Phil Boise’s GreenCare for Children program, which currently endorses 60 providers in Santa Barbara County and another 15 nationwide, and Indiana’s 5-Star Childcare program, which endorses 80 providers within that state.

In addition to the outreach and education accompanying each of these programs, a number of independent efforts are under way. Among them, Vickie Leonard, who previously worked with Bradman on an educational campaign promoting IPM,^23^ and Carol Westinghouse,^24^ program manager for the Vermont-based nonprofit Informed Green Solutions, are collaborating on a green cleaning, sanitizing, and disinfection toolkit for child care centers that they will begin to distribute nationwide in June 2013.

## Complex Regulatory Environment

Yet voluntary programs alone aren’t enough to fully protect young children, Leonard says. “I think it’s an environmental justice issue, because parents in the middle and upper classes are much more aware of these issues and tend to push for safer environmental practices and products. They can choose where they place their children; poor families usually cannot. For that reason, it seems you’ve got to legislate basic environmental safety in child care.”

Laws and licensing requirements designed specifically to protect environmental health at child care centers are scarce, Leonard says. Environmentally safer cleaning and pest-management guidelines introduced at schools are rarely applied to child care centers—and even then it’s often in a delayed or piecemeal fashion. State-level child care licensing requirements are particularly slow to change, as they’re often reviewed only once every three to five years, says Paul.

Still, a number of positive models exist. California’s HSA has been expanded to include early childhood education centers, adding new pest-control requirements such as parental notification of pesticide applications, warning signs, record keeping at child care centers, and pesticide use reporting by pest-control businesses that operate at the centers.^25^ The HSA also promotes the use of IPM in child care centers.^26^ However, Leonard points out, the law lacks an enforcement mechanism.

Since 2001 Vermont has banned pesticide use at child care facilities except as a last resort—defined loosely as “when other pest prevention and control measures fail”^27^—and has required written notification to both parents and staff prior to any planned application.The state may also consider inclusion of green cleaning standards in its licensing requirements, which are currently being revised, Westinghouse says. A 2012 analysis of environmental health policies throughout New England by students at the University of Vermont’s College of Medicine found the state’s child care regulations to be “exemplary by comparison to other northeastern states,” but still having room for improvement in pesticide notifications, ventilation standards, lead exposure screenings, and radon and VOC regulations, among other areas.^28^

Pennsylvania, meanwhile, mandates the use of nontoxic arts and crafts materials in child care centers.^29^ The state also is developing a new outreach and training program called the Early Childhood Education Healthy & Green Initiative,^30^ says state Department of Public Welfare spokeswoman Donna Morgan.

Child care–specific regulations on the national level are unlikely to be forthcoming, says Kathy Seikel, who leads the U.S. EPA’s efforts to promote environmental health in child care settings.^31^ “There aren’t any universal policies for environmental health in child care facilities,” she says. “One of the challenges we face is that we don’t have regulatory authority over child care. So we focus on outreach and education by providing technical assistance and advice on best practices.”

General laws limiting formaldehyde and flame retardants could go a long way toward protecting infants and toddlers against exposure, Bradman says. But as his study showed, it can take years to see reductions in indoor levels of these toxics due to extended phase-in periods and the long-term time frame of furniture and building material replacement.^10^

Both Bradman and Paul support increased regulation yet remain wary of placing excessive hardships on cash-strapped centers. “There’s got to be a way of working with child care providers and the industry … so that it doesn’t impose new burdens,” Bradman says. As Leonard notes, efforts to introduce bleach alternatives that are less hazardous to human health and the environment have been stymied largely because the new products are more expensive than bleach, which is cheap and readily available.

One way or another, the child care industry appears on the verge of a breakthrough in environmental health. “I think those of us working on this now are five years ahead of the game,” Boise says. “I think at some point there will be a collective recognition. Everybody will get it at the same time, and they’ll look to what the existing resources are, and then we’ll be there.”

Van Groenou advises a big-picture view. “A piecemeal approach does not bring about lasting results,” she says. “Early childhood centers must think of both indoor and outdoor environments that affect children, and educate children and parents to bring about real change through examples of action taken by the center.” The use of chemicals and their effects on children is “of course essential to look at,” she says, “but the early childhood education industry needs to think in broader terms of ecological impacts and participation in decisions that are sustainable.”

## Suggested Resources

Eco-Healthy Child Care® Factsheets http://www.cehn.org/ehcc/factsheets

Integrated Pest Management: A Toolkit for Early Care and Education http://cerch.org/research-programs/child-care/integrated-pest-management-a-toolkit-for-early-care-and-education-programs/

Go Green Rating Scale for Early Childhood Settings http://www.gogreenratingscale.org/

Coming soon—The Green Cleaning, Sanitizing, and Disinfecting Toolkit presents practical information on how to keep child care environments clean and safe while protecting young children and staff from infectious diseases. http://www.informedgreensolutions.org/?q=publications/green-cleaning-toolkit

Coming soon—Transpare™ is an online registry to help purchasers choose cleaning products based on environmental, safety, and health attributes. http://www.transpare.com
